# Feasibility of Electronic Health Information and Surveillance System (eHISS) for disease symptom monitoring: A case of rural Ghana

**DOI:** 10.1371/journal.pone.0197756

**Published:** 2018-05-24

**Authors:** Aliyu Mohammed, Konstantin Franke, Portia Boakye Okyere, Johanna Brinkel, Axel Bonačić Marinovic, Benno Kreuels, Ralf Krumkamp, Julius Fobil, Jürgen May, Ellis Owusu-Dabo

**Affiliations:** 1 Kumasi Centre for Collaborative Research in Tropical Medicine (KCCR), Kumasi, Ghana; 2 University Medical Centre Hamburg-Eppendorf (UKE), Hamburg, Germany; 3 School of Public Health (GSPH), Accra, Ghana; 4 National Institute for Public Health and the Environment (RIVM), Bilthoven, Netherlands; 5 Bernhard Nocht Institute for Tropical Medicine (BNITM), Hamburg, Germany; University College London, UNITED KINGDOM

## Abstract

**Introduction:**

The current surge of mobile phone use in many African countries creates the opportunity to provide caregivers with limited access to the health care system with vital health recommendations. At the same time such communication system can be utilised to collect tempero-spatial data on disease symptoms.

**Objective:**

We assessed the feasibility of an mHealth system among caregivers with children under-five years, designed as a health information and surveillance tool in a rural district of Ghana.

**Methods:**

A mobile phone-based *electronic health information and surveillance system* was piloted from February to December 2015. Toll-free numbers were provided to 1446 caregivers, which they could call to receive health advice in case their children showed disease symptoms. The system was setup to evaluate the illness of a sick child. Symptoms reported via the system were evaluated and compared to clinician’s report after follow-up. Cogency of the reported symptoms was assessed using Cohen’s kappa coefficient.

**Results:**

A total of 169 children with disease symptoms were identified based on phone calls from caregivers. The predominant reported symptoms were fever (64%; n = 108), cough (55%; n = 93) and diarrhoea (33%; n = 55). Temporal pattern of symptomatic cases revealed a peak saturation in the month of September, with fever registering the highest number of symptoms observed. Reported symptoms and clinician’s report revealed a very good agreement for fever (95%, kappa = 0.89); good for diarrhoea (87%, kappa = 0.73) and moderate for cough (76%, kappa = 0.49).

**Conclusion:**

This pilot concept, has demonstrated the practicality of using mobile phones for assessing childhood disease symptoms and encouraging caregivers to seek early treatment for their children if needed. The strategy to use mobile phones in disease surveillance and treatment support is a promising strategy especially for areas with limited access to the health care system.

## Introduction

Worldwide, 5.9 million deaths (43 per 1,000 live births) in children under-five years are due to preventable diseases such as malaria, pneumonia and diarrhoea, with the African region alone accounting for 47% of these deaths [1]. In Ghana, progress in reducing child mortality has not been sufficient, decreasing from 80 per 1,000 live births in 2010 to 60 per 1,000 live births [2], which was beyond the unachievable Millennium Development Goal target of 40 deaths per 1,000 live births and still remains a far cry from the Newborn Strategy and Action Plan (2014–18) goal of reducing newborn deaths to 21 per 1,000 live births [3]. Malaria remain a major public health concern, with 36% of children (6–59 months) affected in 2014 [2].

However, early detection and effective treatment of malaria is known to save lives by seeking early treatment [4]. These efforts are highly constrained by weak health systems in rural areas, as well as factors such as travel-distance to health facilities, inadequate drug stocks at health facilities and lack of money to pay for services particularly in sub-Saharan African countries [5]. Failure to recognize signs and symptoms of childhood illness by caregivers have been attributed to delay in seeking care [6]. In an effort to reduce the burden of disease and provide appropriate care, Ghana adopted the Integrated Management of Childhood Illness (IMCI) policy. The IMCI guidelines require that sick children who show general danger signs such as inability to feed, incessant vomiting, unconsciousness and convulsions, be managed quickly and referred immediately after pre-referral treatment [7].

In the meantime, during the last two decades, there has been a surge of over 27 million mobile phone users in Ghana [8]. Mobile health (mHealth), the use of mobile electronic devices for public health purposes, holds the potential to overcome several challenges that plague the healthcare system. It has been used extensively in many health programmes to improve interaction between patients and the health system [9]. mHealth has been exploited in health programmes including chronic disease management [10], medication compliance [11], substance abuse [12], mental illness [13] and post-discharge monitoring of patients [14]. A consortium of medical, public health and mHealth experts from Ghana, Netherlands and Germany, in collaboration with the Ghana Health Service, piloted an Electronic Health Information and Surveillance System (eHISS), to evaluate the feasibility of using a mobile phone-based technology for monitoring symptoms of childhood illnesses and to encourage caregivers to seek early and appropriate healthcare. Based on the IMCI guidelines [7] the system is able to interact with caregivers via an Interactive Voice Response (IVR) algorithm and provide first treatment recommendations. The IVR is an automated mobile phone-based technology deigned to allow a caller to dial into a computer system over a telephone line and access a service running on the computer. The caller may then interact with and receive voice information from the service. So far, little is known about the feasibility and utilisation of the IVR-based application among caregivers of children under-five years of age in a malaria endemic country like Ghana. This paper therefore aims to assess the feasibility of a symptom-oriented mHealth system among caregivers of children under-five in a rural district of Ghana.

## Materials and methods

### Study area and participants

This pilot study was conducted from February 2015 to December 2015 in the Agogo sub-district of the Asante Akim North Municipal District, located in the Ashanti Region of Ghana. It has a population of approximately 45,870, who mainly engage in small-scale farming. The Agogo Presbyterian Hospital (APH), a local district hospital, provides health services to the entire population and also serves as a referral centre for other hospitals in the region. According to the district health annual report, the commonest childhood disease symptoms recorded among children under-five in the study area were fever, diarrhoea and cough. The eHISS system was piloted within the hospital catchment area. Caregivers of children under-five who could fluently speak the local language (Twi) were recruited to partake in the study.

### mHealth application

**Fig 1:**
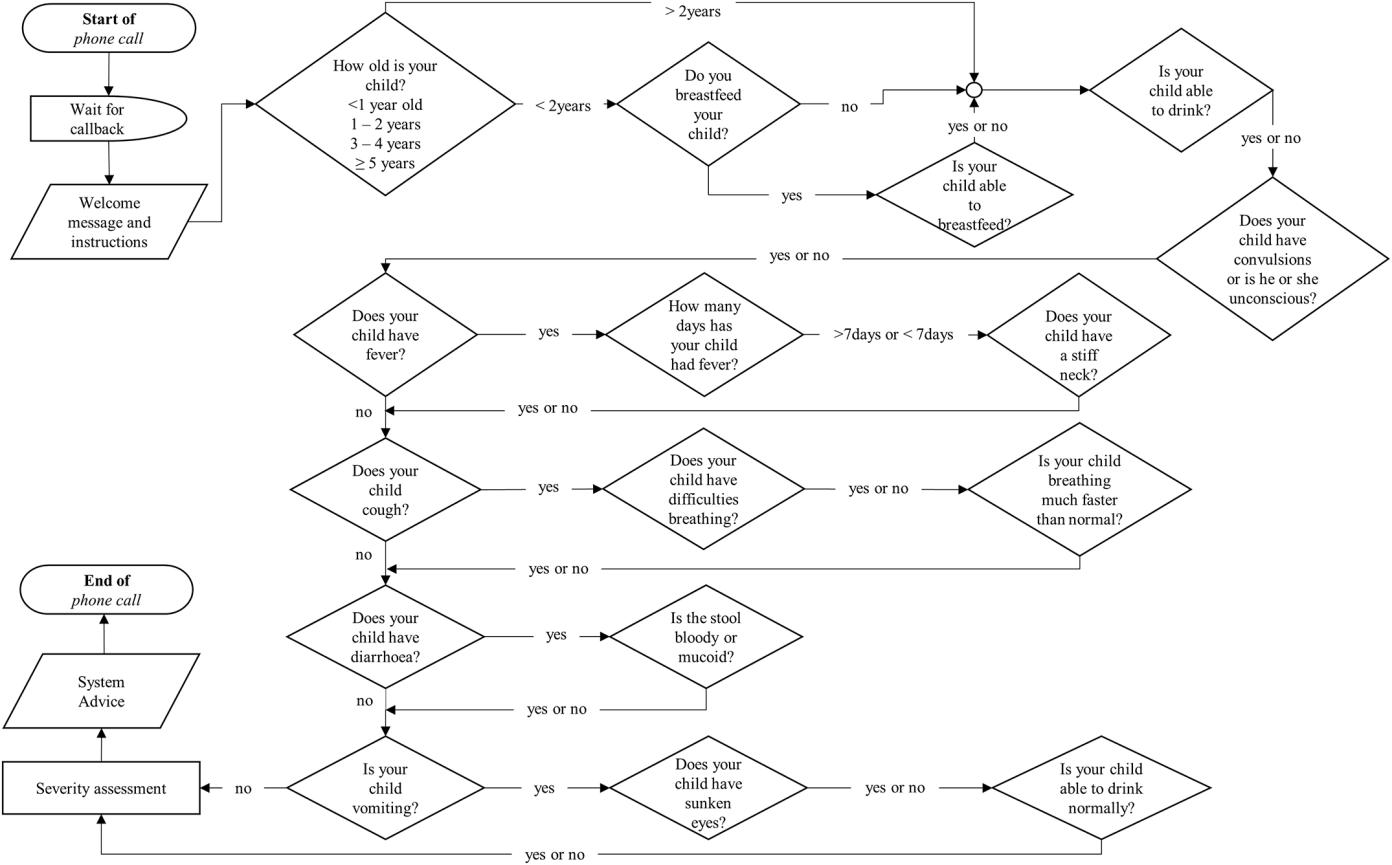
Flowchart of the IVR system algorithm. The arrows show the flow of the questions, with all questions asked irrespective of the response provided in the previous question. The system provides advice at the end of the call based on the combination of symptoms indicated.

Developed and operated by a Ghana-based private company called Viamo, this mHealth system was designed to assess symptoms of sick children and provide health advice to caregivers through mobile phones. Caregivers of sick children were provided with tailor-made health information after answering several sequential questions through an IVR. The automated system was based on a clinical algorithm (Fig 1), so that after answering a set of questions, data on current disease symptoms and geo-location were gathered. The clinical algorithm was based on the decision trees of the IMCI [7], and local assessment of clinician’s report and recommendation. The clinical algorithm and its translation was developed over a period of 2 years by experts consisting of clinicians, public health experts, epidemiologists/biostatisticians and communication researchers. After placing a call, the system automatically ends it and calls back. After a brief introduction, the system provided instructions for the caregivers to give responses by mostly pressing ‘1’ for a ‘yes’ response or ‘2’ for a “no” response on their mobile phones. The first set of questions were asked to assess whether the child had any ‘danger sign’ such as inability to breastfeed or drink or unconscious and had convulsions, taking into consideration the age of the child. The next set of questions were asked to identify the common symptoms of childhood illness including fever, cough and diarrhoea. Further questions were only asked to assess the severity of the child’s condition if the caregiver responded ‘yes’ to the previous question. A caregiver was required to answer all questions asked by the system, so that all symptoms could be captured, and at the end provided with advice after symptom assessment. This advice was based on the level of severity of a symptom or combinations of symptoms—where ‘A’ denoted severe, ‘B’ moderate, ‘C’ mild and ‘O’ none (see S1 Table for detail description of each category). Where a child fell in more than one category after answering all questions, priority was given to the highest category, and the appropriate advice given. As indicated in the IMCI guideline, all danger signs such as inability to breastfeed or drink, unconscious or convulsions and other combinations of symptoms were all classified as severe by the system. All calls that fell in category ‘A’ were deemed as requiring emergency treatment, and consequently, caregivers were advised by the system in their local dialect (‘Twi’) to take their sick child to the nearest health facility immediately. Similarly, symptoms that fell in the ‘B’ category were deemed to require causal treatment, hence advised by the system to take the sick child to the nearest hospital within 24 hours; and symptoms within the ‘C’ category advised to provide home treatment and take child to the nearest health facility if symptoms persist. The home treatment included the provision of Oral Rehydration Salt (ORS) or paracetamol syrup, depending on the symptom reported. On the other hand, symptom reports that fell in the ‘O’ category meant the system could not assess the underlying cause of illness. In such situations, caregivers were advised to take the sick child to the nearest health facility for further assessment in order to ascertain the condition of the child.

Individuals who access the system for health advice are indirectly reporting disease symptoms, which are recorded by the system. These data, along with location and time information are automatically stored and available for immediate analyses. Due to the data protection regulations by the National Communications Authority the phone companies did not provide location data from phone calls. Thus, the study was conducted with registered users only, who provided their phone numbers and coordinates of their current residence.

### Sample size

We estimated the sample size with an assumed ownership of mobile phone by caregivers of 50% and 5% case report of fever symptoms within the study population. Therefore, with a default alpha of 0.05 and a power of 80% and taking cognisance of non-response rate of approximately 20%, we arrived at a sample of 1446. This would enable us to oversample and detect at precision level of 5%.

### Data collection procedures

This study had two major phases, namely (a) a three months testing phase and (b) an eight months implementation phase. During the testing phase, only caregivers who personally owned a mobile phone were registered (details such as phone number, name and location recorded into the system) and this was limited to caregivers who were attending Child Welfare Clinic (CWC) located in the communities. A total of 433 caregivers were recruited. The objective of the testing phase was to test the usability of the system by registered caregivers, system’s ability to provide appropriate advice and assess caregivers’ ability to correctly follow the advice of the system. In the implementation phase, a total of 1013 caregivers were registered.

### Recruitment of study participants

Caregivers were recruited from 36 different communities with a functional mobile network located within the APH catchment area. Caregivers received a toll-free phone number and instructions on how and in which situation to access the system using their mobile phones. They were given the opportunity to use their phone or that of a relative. All registered caregivers at the point of recruitment were briefly trained by dummy demonstration about the use of the system. Using a structured questionnaire, socio-demographic characteristics including age, gender, marital status, education, religion, ethnicity and mobile phone ownership, as well as mobile phone numbers, experience with the use of IVR, name of community and Global Positioning System (GPS) coordinates of the communities were recorded at the point of recruitment.

After seeking permission from the administrative head and the health authority of the study area, eHISS was actively advertised for three months (twice in a week) during the implementation phase with the assistance of community health volunteers through public information centres and social gatherings.

### Follow-up procedures

**Fig 2:**
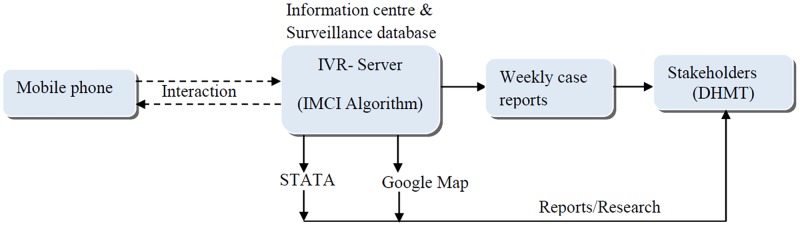
Schematic overview of the eHISS system.

During the study period, all system-recorded calls were followed up within 2 days in order to evaluate the health status of the sick children and the action taken by caregivers. Records of children who were taken to the nearest hospital were retrieved to identify symptoms recorded by the clinicians. Intermittent contact was made with District Health Management Team, a team responsible for monitoring disease occurrence within communities and providing basic medical treatment. They were given weekly reports of emerging symptoms of childhood illness development following caregiver-IVR interaction including the spatial development of reported symptoms generated using google map (Fig 2).

### Data management and statistical analysis

Data recorded during follow up were entered in Access database. Similarly, data obtained from the system were extracted in csv format and exported to STATA 12 (STATA Corp., College Station, Texas, United States) for analysis. Percentage agreement between data obtained from the IVR system and the clinicians was calculated for reported symptoms such as fever, cough and diarrhoea. This was done for 28 caregivers whose IVR reports were sufficient and medical record of reported sick child available. Cohen’s Kappa coefficient (k) was calculated to test for the percentage agreement and the corresponding 95% confidence interval. The values of p < 0.05 were considered significant. Kappa values were interpreted according to Altman’s guidelines [15]; values <0.20 = poor agreement, 0.21–0.40 = fair, 0.41–0.60 = moderate, 0.61–0.80 = good and 0.81–1.00 = very good.

### Ethical considerations

Ethical clearance for the study was obtained from the Kwame Nkrumah University of Science and Technology (KNUST)/Komfo Anokye Teaching Hospital (KATH) Committee on Human Research, Publications and Ethics (CHRPE/AP/278/14). In addition, clearance and permission was obtained from the Director of Health Administration of the Asante Akim North Municipal District. Permission from the respective leaders of the communities was also sought. Written informed consent was obtained from the caregivers and assent from their children.

## Results

### Socio-demographic characteristics of registered respondents

The mean age of registered caregivers was 30 (SD = .20) years, 97% (n = 1401) were female, 65.2% (n = 942) of them were married. Furthermore, 49% (n = 708) had Junior High School education, 85% (n = 1242) were Christians, 68% (n = 980) belonged to the ethic group of Akans, 70% (n = 1008) owned a mobile phone, and 73% (n = 1047) had no prior experience with IVR applications (Table 1).

**Table 1:** Socio-demographic characteristics of registered caregivers and experience with IVR (N = 1446).

Variable	n	%
Age group (years)		
≤20	201	14.0
21–30	721	50.0
31–40	408	28.0
41 +	116	8.0
Total	1446	100.0
Age; mean (SD)	29.9	0.20
Sex		
Male	45	3.0
Female	1401	97.0
Total	1446	100.0
Marital status		
Married	942	65.2
Single	321	22.2
Cohabiting	171	11.8
Divorced/Widow	12	0.8
Total	1446	100.0
Education level		
No formal education	233	16.1
Primary	207	14.4
Junior High School	708	49.1
Senior High School	203	14.1
Tertiary	91	6.3
Total	1442	100.0
Religion		
Christianity	1242	85.1
Islam	190	13.1
Traditionalist	14	1.0
Total	1446	100.0
Ethnicity		
Akan	980	67.8
Ewe	35	2.4
Kusaasi	108	7.5
Grussi	60	4.2
Frafra	38	2.6
^a^Other	225	15.6
Total	1446	100.0
Mobile Phone Ownership		
Self	1008	69.7
Partner	342	23.7
Relative	96	6.6
Total	1446	100.0
Experience with IVR		
Yes	389	30.0
No	1054	73.0
Total	1443	100.0

^a^Other includes Ga, Krobo, Gruma, Dagati, Fulani, Mamprusi, Chamba

**Fig 3:**
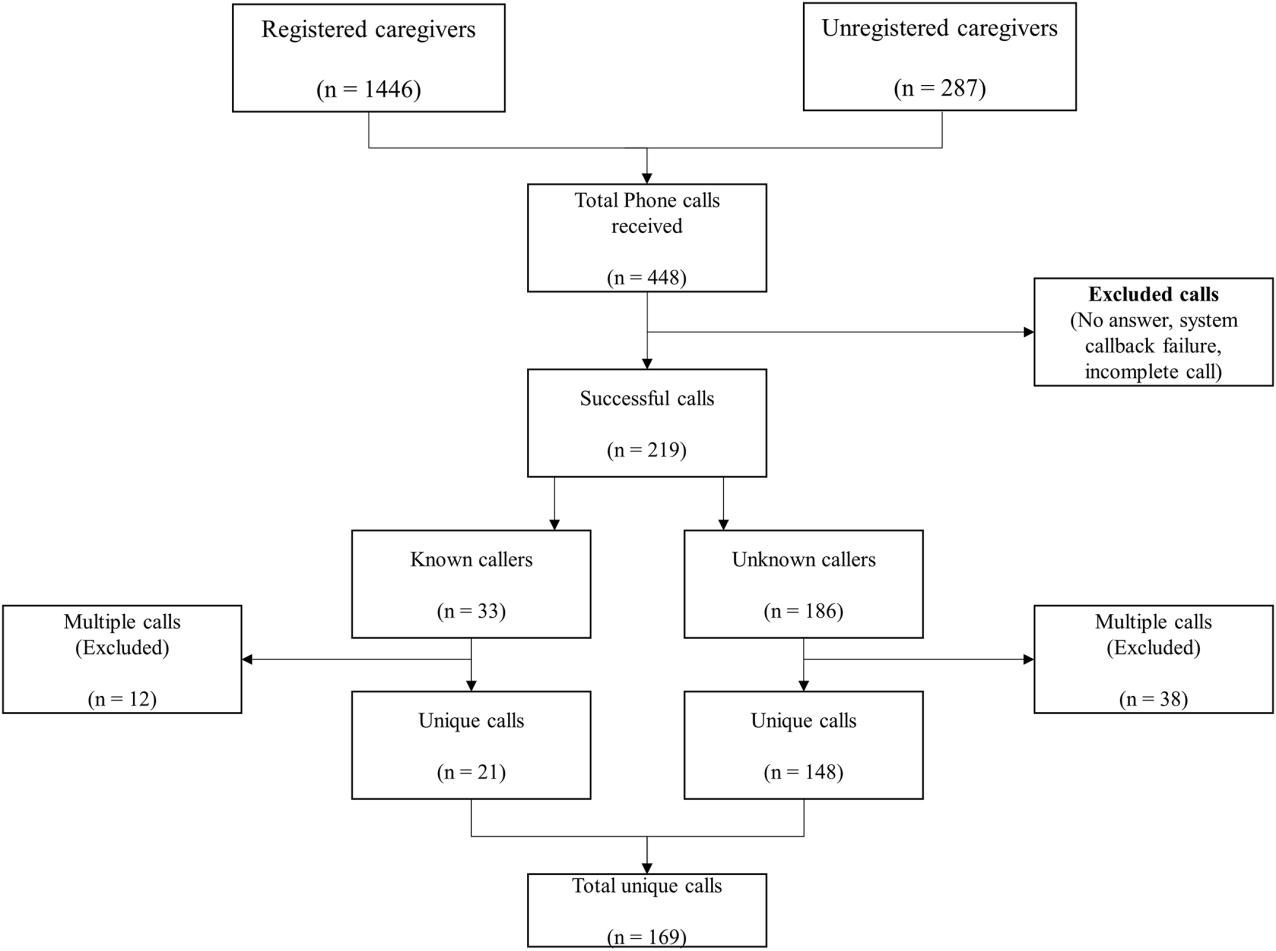
Flowchart of calls made to the eHISS system and individuals included into the study.

Of the 448 calls received from both registered and unregistered users, 229 calls were excluded mainly because the callers did not specify any symptom. Callers who reported at least one symptom were included in the analysis. All multiple calls were counted as one and added to the unique-call total, for both registered and unregistered callers, adding up to an overall 169 unique calls (Fig 3).

Based on the symptoms indicated by the caregiver, treatment recommendation provided by the system predominantly fell within the emergency category ‘A’ (55%, n = 93). Over forty percent (41%, n = 69) who fell within category ‘B’ and ‘C’ were advised to take their children to the nearest health facility after providing some level of care. Less than 5% of the calls which fell in the ‘O’ category could not be provided with useful advice, thus asked to seek advice for the unknown symptom. Of the 28 caregivers who were successfully followed-up by phone, 68% (n = 19) reported taking their children to the hospital and 11% (n = 3) providing home treatment as recommended by the system. One-fifth (21%, n = 6) however, did not take any action.

In total, 49% completed incoming calls and 51% unsuccessful calls were recorded by the system. The majority (85%, n = 144) of the caregivers selected Twi as their preferred language. The mean duration of time spent by caregivers interacting with the system was 4 minutes (SD = 1.9). Assessment of similarity of symptoms reported by caregivers and clinician’s report revealed a very good agreement for fever (95%, kappa = .89, *P* < 0.001) and good for diarrhoea (87%, kappa = .73, *P* < 0.01) and moderate for cough (76%, kappa = .49, *P* < 0.05) (Table 2).

**Table 2:** Cohen’s kappa coefficient for inter-rater agreement between data obtained from the IVR system and clinician’s report.

Variable				Agreement(%)	Cohen kappa coefficient	95% CI	P-value
IVR System	Clinician						
**Fever**	Fever		Total	95.0	0.89	0.68–1.00	<0.001
	Yes	No					
Yes	11	1	12				
No	0	7	7				
Total	11	8	19				
**Cough**	Cough		Total	76.0	0.49	0.05–0.92	0.02
	Yes	No					
Yes	9	2	11				
No	2	4	6				
Total	11	6	17				
**Diarrhoea**	Diarrhoea		Total	87.0	0.73	0.39–1.00	0.002
	Yes	No					
Yes	6	1	7				
No	1	7	8				
Total	7	8	15				

**Fig 4:**
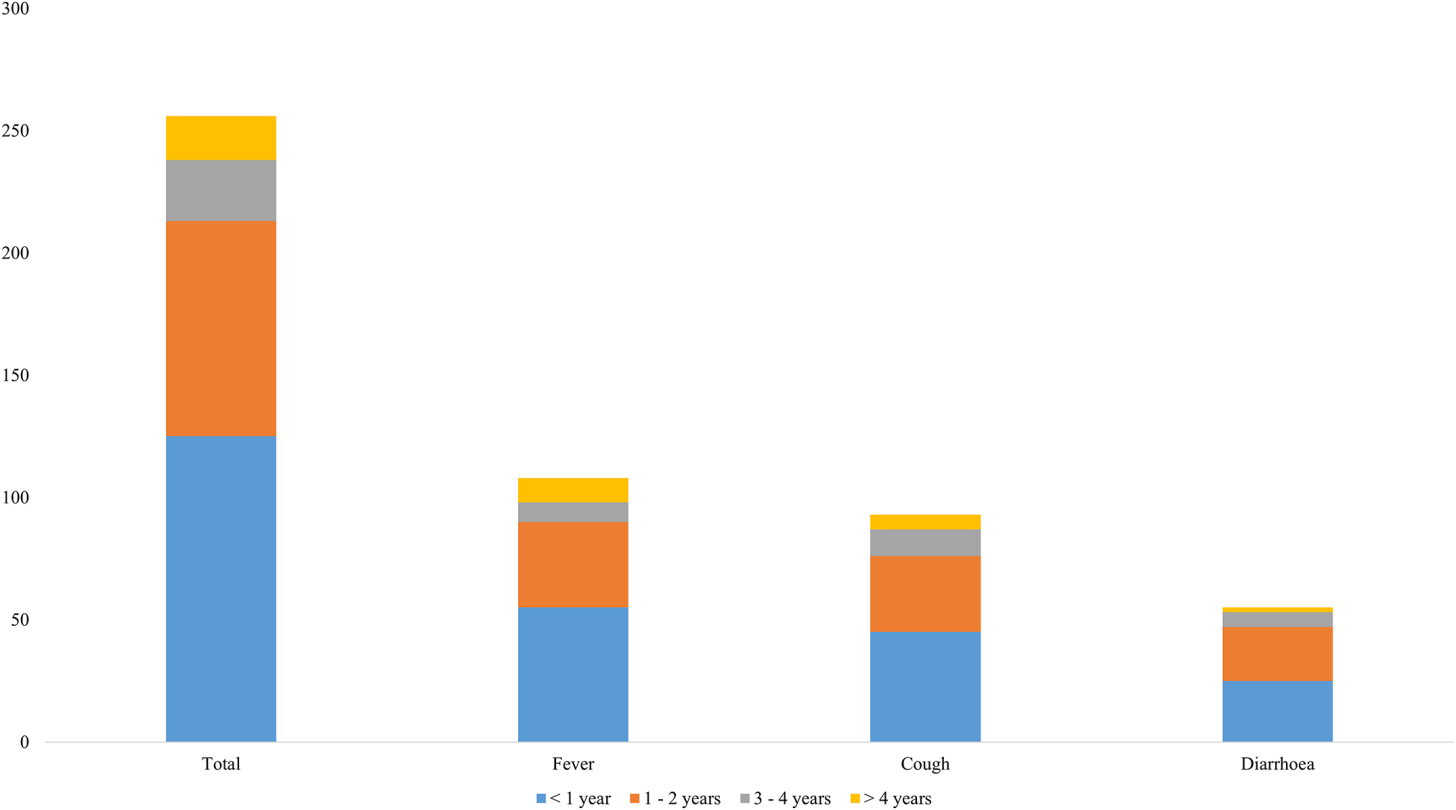
Symptoms reported by age of child.

The total number of symptoms accrued was estimated for communities involved in the study. The highest numbers of symptoms were recorded in communities such as Bontodiase (n = 151), Newtown (n = 138), Oboase/Mponisa (n = 205) and Gyedim (n = 97), with (n = 9) in Kusibo. The most reported symptoms were fever (64%; n = 108) and cough (55%; n = 93) while the least symptom was diarrhoea (33%; n = 55). Of the 169 children who were reported ill, more than half (52%; n = 88) were less than 1 year old, while less than 10% were 2–4 years (9%; n = 15) and at least 5 years old (8%; n = 14) as shown in Fig 4. Majority (86%, n = 145) of the caregivers also reported at least one danger sign.

**Fig 5:**
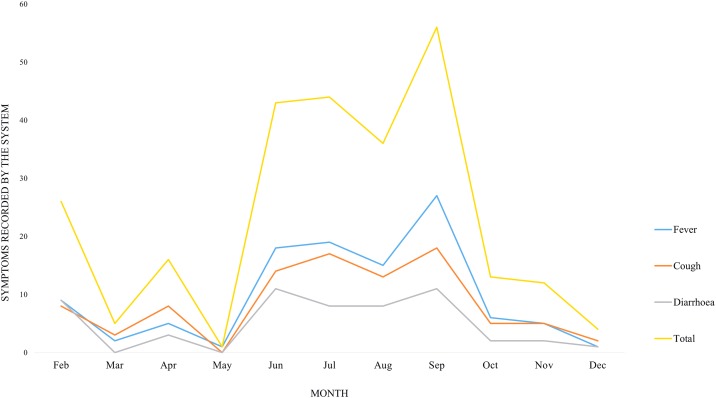
Temporal patterns of symptoms reported. Temporal pattern of symptoms identified by the system based on phone calls from caregivers. Symptom saturation was observed in May-October.

Fig 5 shows the distribution of the 169 symptomatic cases identified throughout the study period, with the highest number of symptoms reported in the month of September. Fever was the predominant symptom reported in the peak period, with 27 cases observed. The temporal pattern of symptomatic cases identified among registered and unregistered users of the system.

## Discussion

Our study illustrates how a mobile phone-based electronic health information and surveillance system can be deployed to provide useful health advice, while at the same time providing information on potential disease clusters in a rural district. It was set up to test the potential to detect outbreaks by analysing spatiotemporal data on disease symptoms reported by service users.

Studies involving the use of IVR have reported varying access rates—ranging from 12.5% to 63.4% [16–18]. In the current study, the 11 month period yielded 169 calls with unique disease symptom reports of sick children from both registered and unregistered callers. Considering the number of registered caregivers and the universal access to mobile phone, the number of calls recorded by the system was generally low. This could be due to a number of reasons, including children not falling ill, lack of motivation and trust in a computer-based system, first-time use and lack of experience with the IVR system. Although the education level of the caregivers was generally low, this could not have accounted for the low report rate as some studies have found no association between IVR use and education level [18,19]. Nonetheless, a larger proportion of the calls recorded from the system came from unregistered community members who received no prior instructions on how to use the system. This clearly suggests that employing good marketing and advertisement strategy (e.g, at social gatherings or community information centres) for raising awareness could increase the number of people utilizing the system. As expected, most users preferred to use the system with the local language (Twi), which may have been easier for them to understand and report symptoms effectively as observed in an earlier study conducted by Piette and colleagues [19].

Other studies have documented the usefulness of using Short Message Service (SMS) to increase attendance to health care services [20] and adherence [21,22]. These studies have shown that, SMS reminders increase the likelihood of clinic appointments for general illnesses [23,24]. In our study, however, the system was designed to give advice depending on the severity of the symptom whether to provide home treatment or seek immediate treatment at the nearest health facility for caregivers who reported at least one symptom. This could help lay people care for their sick children and to triage them to the healthcare system, potentially reducing the likelihood relying on self-treatment and drug vendors which could result in misdiagnosis and incorrect choice of drugs [25] and subsequently, development of adverse drug reactions and dangerous drug interactions [26]. As one of the key measure of performance, the capacity of the system to encourage caregivers to seek early treatment was assessed. Although during the study period not all actions taken by all caregivers who accessed the system could be ascertained, caregivers who were successfully followed up by study team revealed that majority of them took their sick children to the nearest health facility for appropriate treatment. This suggests that the system could be deployed to tackle the current existing problem of about 44% of children who experience fever and not taken to a health facility for appropriate treatment in Ghana [2]. Caregivers who also reported at least one danger sign were recommended to seek immediate medical treatment at the appropriate source. This also suggests that, caregivers who may have been oblivious of the extent of the illness of the child and may not have otherwise acknowledged them as danger signs were accurately advised by the system to seek immediate and appropriate care at the nearest hospital, potentially reducing delayed treatment. In the future, the mobile phone-based system could be linked to a medical personnel at post in order to encourage timely reporting of symptoms. As a potential behaviour change tool, the system could also be used primarily to improve healthcare utilization and maternal and child health services such as antenatal care and immunization coverage [27].

Using a web-based mobile phone technology, Meankaew and others (2010) implemented a disease and treatment monitoring of malaria (DTMM) module in Thailand to assess its effectiveness. The follow-up rates were found to be fairly high, with the system capturing 534 patients with malaria [28]. Though similar to the study conducted in Thailand, our study solely relied on the unilateral and self-reported symptoms by caregivers, with system-user interaction monitored in real time. Temporal pattern of symptoms detected by the system employed in our study showed that symptoms were reported between May and October. Fever was found to be the most frequent symptom observed, steadily increasing from May to September. This finding is consistent with the temporal pattern of malaria prevalence within the study area, where malaria is endemic and known to be the number one cause of fever [29] during the month of June to August [30].

As an important system component, the validity of the IVR system reports provided by the caregivers were validated using Cohen’s Kappa coefficient. Although the study revealed varying concordance for all reported symptoms, percentage agreement between the IVR report and the medical record ranged from very good (0.89) to moderate agreement (0.48). This is consistent with a study conducted to validate the internal consistency of IVR and internet self-reports with clinician’s report among addiction patients using an Addiction Severity Index (ASI) [31] and earlier reliability and validity studies of IVR system [19,32]. This demonstrates the feasibility and reliability of using IVR for reporting symptoms in near-real time which also provides cues for diagnosis. However, additional work is needed to identify factors that affect IVR reporting quality in order to increase response rate for reported symptoms. The question-completion rate of 49% revealed in our study was generally low compared with about 90% completion rate achieved in the study involving addiction clients [31]. The difference in the completion rate is most likely because of the study population, as substance abuse persons may feel less judged conveying their substance use to a computer than a person. Comparatively, a major motivation factor for caregivers to access an IVR could be compelled by the child’s condition.

### Limitations

Although the eHISS pilot study provided data for monitoring symptoms of childhood illnesses, a number of challenges were encountered during its implementation. Therefore, some limitations should be considered when interpreting the results of the study. Physical barriers such as unstable mobile network in some communities, low ownership of mobile phones, ineffective marketing strategy and hard-to-reach communities could have accounted for the low number of calls received during the study period. However, a major challenge was data quality- incompleteness. This implies that additional efforts are required to completely clean the data, adding to the time taken data extracted from the system to be ready for use. Additionally, all incoming calls that could not specify at least one symptom were excluded from the case analysis, indicating loss of potential cases. The absence of Geographic Information Systems (GIS) data also made it impractical to perform cluster analysis of diseases symptoms. Also, all reported symptoms could not be validated to ascertain whether caregivers were reporting “true” symptoms of their children.

## Conclusion

As a pilot concept, this study has demonstrated the practicality of using mobile phones for monitoring childhood disease symptoms and encouraging caregivers to seek appropriate and early treatment for their children. However, further studies are needed to assess the impact of the system on health outcomes and its capacity to provide spatial monitoring of infectious diseases such as cholera, HIV, tuberculosis etc. as a surveillance tool for disease control efforts.

## Supporting information

S1 Table Level of severity of reported illness and recommendation provided by the system. (DOCX)
