# Supplementary material for: Feasibility of Electronic Health Information and Surveillance System (eHISS) for disease symptom monitoring: A case of rural Ghana
Source: PLoS One. 2018 May 24;13(5):e0197756. doi: 10.1371/journal.pone.0197756 (PMC5967752; doi:10.1371/journal.pone.0197756)
Supplement: S1 Table — (DOCX) [file pone.0197756.s001.docx]

S1 Table. Level of severity of reported illness and recommendation provided by the system

| **Category** | **Symptom assessment** | **Symptom combinations** | **System Advice** | **Clinician’s report** |
| --- | --- | --- | --- | --- |
| A’  Severe | Unable to breastfeed | Unable to breastfeed +/- Any symptom | **Requiring emergency treatment:**  “Take your child to the nearest health facility immediately!” | **Emergency treatment:**  For example intravenous fluids, intravenous antimalarial drugs or hospitalization |
|  | Unable to drink | Unable to drink **+/-** Any symptom |  |  |
|  | Unconscious or Convulsions | Unconscious or Convulsions **+/-** Any symptom |  |  |
|  | Severe febrile disease | Fever + [ >7 days or <7 days] + stiff neck |  |  |
|  | Severe Respiratory Tract Infection | Cough + difficult breathing + breathing faster than normal |  |  |
|  | Gastrointestinal – Infection with severe dehydration | Diarrhoea **+** vomiting + [sunken eyes and/or not able to drink normally] |  |  |
| B’  Moderate | Febrile disease | Fever + [ >7 days or <7 days] – stiff neck | **Requiring causal treatment:**    “Take your child to the nearest health facility within 24 hours!” | **Causal treatment:**  For example antibiotics, antimalarial (oral form) or follow-up |
|  | Moderate Respiratory Tract Infection | Cough + difficult breathing or breathing faster than normal |  |  |
|  | Dysentery | Diarrhoea + bloody or mucoid stool |  |  |
|  | Gastrointestinal – Infection with some dehydration | [Diarrhoea and/or vomiting] + sunken eyes - not able to drink normally] |  |  |
| C’  Mild | Mild Respiratory Tract Infection | Cough – [difficult breathing **+** breathing faster than normal] | **Requiring home care:**  “Treat your child at home and assess disease progression carefully!” | **Symptomatic treatment:**  For example nasal drops or paracetamol or follow-up not needed |
|  | Gastrointestinal – Infection with no dehydration | [Diarrhoea and/or vomiting] – [sunken eyes + not able to drink normally] |  |  |
| O’  None | None-categorised symptom combination | No positive symptom indicated | **Requiring further assessment:**  “System unable to determine the underlying cause of your child’s condition. Take child to the nearest health facility for further assessment!” | **Treatment provided based on type of disease diagnosed.** |
